# Play Behavior Varies with Age, Sex, and Socioecological Context in Wild, Immature Orangutans (*Pongo* spp.)

**DOI:** 10.1007/s10764-023-00414-2

**Published:** 2024-01-20

**Authors:** Julia A. Kunz, Sonja S. Falkner, Fikty Aprilinayati, Guilhem J. Duvot, Marlen Fröhlich, Erik P. Willems, Sri Suci Utami Atmoko, Carel P. van Schaik, Caroline Schuppli, Maria A. van Noordwijk

**Affiliations:** 1 Department of Evolutionary Anthropology, University of Zurich, Zurich, Switzerland; 2 Institute of Evolutionary Science of Montpellier (ISEM), University of Montpellier, CNRS, IRD, Montpellier, France; 3 Department of Biology and Primate Research Center, Universitas Nasional, Jakarta, Indonesia; 4 Palaeoanthropology, Institute for Archaeological Sciences, Department of Geosciences, University of Tübingen, Tübingen, Germany; 5 Comparative Socioecology Group, Max Planck Institute of Animal Behavior, Constance, Germany; 6 Department of Evolutionary Biology and Environmental Studies, University of Zurich, Zurich, Switzerland; 7 Development and Evolution of Cognition Research Group, Max Planck Institute of Animal Behavior, Constance, Germany

**Keywords:** Developmental trajectories, *Pongo*, Play, Social tolerance, Socioecological variation

## Abstract

Play is thought to serve different purposes at different times during ontogeny. The nature and frequency of play are expected to change accordingly over the developmental trajectory and with socio-ecological context. Orangutans offer the opportunity to disentangle the ontogenetic trajectories of solitary and social play with their extended immature phase, and socio-ecological variation among populations and species. We evaluated the frequency of play in 39 immature individuals across two populations (*Pongo pygmaeus wurmbii*, at Tuanan, Borneo, and *P. abelii* at Suaq, Sumatra), age (0–11 years), sex, and social context, using more than 11 500 h of full-day focal observation data. We found independent age trajectories of different play types, with solitary object and solitary locomotor peaking before social play. Social play partners changed during ontogeny, and male immatures were more likely to engage in non-mother social play than females. Overall, social play was more frequent at Suaq than Tuanan, linked to the more frequent availability of partners. Furthermore, per time in association with conspecifics, Tuanan immatures were as likely to engage in social play as their peers at Suaq, suggesting similar intrinsic motivation. Increasing fruit availability correlated with both longer associations and increased social play frequency in the less sociable population of Tuanan, but not at Suaq. Our findings on orangutans support evidence from other species that different play types follow different developmental trajectories, vary with sex, social opportunities, and ecological context. Although drawing functional inferences is challenging, the distinct developmental trajectories reflecting adult sociability and behavioral repertoires may indicate that play serves several, non-mutually exclusive functions during ontogeny.

**Supplementary Information:**

The online version contains supplementary material available at 10.1007/s10764-023-00414-2.

## Introduction

Play can be observed in most vertebrates, and in some invertebrates (Burghardt, 2005, 2015; Fagen, 1981; Zylinski, 2015). Because of its omnipresence and the costs that come with it, play is expected to have an adaptive value, rather than being a by-product or even a maladaptation (Bekoff & Byers, 1981). However, defining play, let alone evaluating its fitness consequences, remains notoriously difficult (Miller, 2017). We use the definition that *“play is repeated, seemingly non-functional behavior differing from more adaptive versions structurally, contextually, or developmentally, and initiated when the animal is in a relaxed, unstimulating, or low stress setting”* (p. 91 Burghardt, 2014). Play is commonly divided into three types: social, solitary object, and locomotor-rotational play (Bekoff & Byers, 1981), and these three play types can be combined within one single bout. Being a heterogenous phenomenon, it is debated whether play types have evolved independently and serve different functions (Burghardt, 1998; Graham & Burghardt, 2010). Empirical studies supporting the numerous theories about the evolution of play behavior remain scarce (Sharpe, 2019).

Play is predominantly witnessed in immature animals and declines in frequency from late infancy to adolescence in mammals (Fagen, 1993), although it is observed among adults in some species (dogs [*Canis familiaris*]: Bauer & Smuts, 2007; bonobos [*Pan paniscus*]: Enomoto, 1990; primates: O’Meara et al., 2015). Play by immature individuals may be a rehearsal for adult behavior (Thompson, 1998), and its nature likely changes with developmental trajectories of different skills (sensu Piaget, 1962). Several non-mutually exclusive theories have been proposed regarding the benefits of play during development, including improved motor (Berghänel et al., 2015; Byers & Walker, 1995), sensory (Fairbanks, 2000), social (Fagen, 1981) or cognitive skills (Pellis & Iwaniuk, 2000), and a general preparation to react to the unexpected (Spinka et al., 2001). Play may have contributed to the evolution of intelligence and behavioral flexibility (Bateson, 2011; Bekoff, 1995; Pellegrini, 2009), and facilitate innovations and their transmission (Pellegrini, 2013; Riede et al., 2021). Accordingly, larger-brained species generally show higher play frequencies as well as more complex sequences of play (Kaplan, 2020; Lewis, 2001; Lewis & Barton, 2004, Lewis & Barton, 2006). In sum, play is likely multifunctional (Pellis et al., 2010).

Supporting the argument that play serves multiple functions, play behavior varies with age, sex, and environmental and social context between and within species and may reflect context- and development-dependent functions and constraints. First, the three play types show different age trajectories with consecutive peaks of solitary locomotor, solitary object, and social play (chimpanzees [*Pan troglodytes*]: Cordoni & Palagi, 2011; vervet monkeys [*Chlorocebus pygerythrus*]: Fairbanks, 2000; gorillas [*Gorilla gorilla*]: Maestripieri & Ross, 2004; meerkats [*Suricata suricatta*]: Sharpe, 2005). Second, sex differences reflect adult sociality and skill repertoires, in that male immatures exhibit higher social play frequencies, particularly rough-and-tumble play, compared to female immatures (humans [*Homo sapiens*]: Lew-Levy et al., 2022; primates: Lonsdorf, 2017; mammals: Marley et al., 2022). Further, immature chimpanzee males have more social partners (Lonsdorf et al., 2014a, b) and show different types of object play compared to females (Kahlenberg & Wrangham, 2010). Third, elevated rates and more sophisticated forms of object play and manipulation are associated with the occurrence of habitual tool use (e.g., chimpanzees versus bonobos: Koops et al., 2015a, b; Myowa-Yamakoshi & Yamakoshi, 2011) and with specific (complex) foraging niches (Bock & Johnson, 2004; O’Hara & Auersperg, 2017). Fourth, play is a costly behavior, and among the first activities to cease when an individual is exposed to stressors (Held & Špinka, 2011; Martin & Caro, 1985). In wild populations, social play frequency varies accordingly with food abundance in some species (Burghardt, 2005; e.g., mantled howler monkeys [*Alouatta palliata*]: del Toro et al., 2019; squirrel monkeys [*Saimiri sciureus*]: Stone, 2008). Finally, the frequency and particularly the quality of social play interactions among immature individuals have been associated with more socially complex societies, and adult social and/or fighting competence (e.g., domestic pigs [*Sus scrofa domesticus*]: Turner et al., 2020). Where this is important, mothers may modify their own behavior to facilitate the socialization of their offspring by associating with suitable partners. This is most apparent in species with high fission–fusion dynamics. Chimpanzee mothers with juvenile offspring, at the age when most play occurs, have been reported to associate more compared to mothers with infants (Williams et al., 2002), and mothers with sons provided them with more play opportunities compared to mothers with daughters (Murray et al., 2014), reflecting male immatures' need to set up social bonds in the light of male-male coalitions for territory defense and hunting in a male philopatric society. In sum, while inferring functions to play behavior in empirical studies may be challenging, play behavior and its different types vary with individual and socio-ecological context, and reflect differences in adult behavioral repertoires in various taxa.

The social system of orangutans (*Pongo* sp.) is characterized by individual-based fission–fusion dynamics (van Schaik, 1999), with mothers spending most time alone with their youngest offspring, and considerable socio-ecological and cultural variation among populations and species (Delgado & van Schaik, 2000; Krützen et al., 2011; van Schaik et al., 2003, 2006; Wich et al., 2012). Due to their exceptionally slow life history and development (a dependency phase of 6 to 9 years: van Noordwijk et al., 2009, 2018; Wich et al., 2009) and large brains (Taylor & van Schaik, 2007), orangutans offer an opportunity to disentangle the trajectories of different play types. Indeed, skill acquisition has been reported to vary depending on species, ecology, and culture (Jaeggi et al., 2008, 2010; Schuppli et al., 2016a, b; Schuppli, van Cauwenberghe et al., 2021b).

Play behavior of orangutans has been studied predominantly in captivity (Jantschke, 1971; Zucker & Thibaut, 1995), and likely differs from behavior in the wild due to (i) the housing in permanent groups, which offers more opportunities for social play, and (ii) the safety of the captive environment, resulting in more terrestrial activity (e.g., Fröhlich et al., 2021). In wild populations, unweaned offspring spent about 15–45% of their time in solitary play but only 0.5–1.5% in social play on average (van Noordwijk et al., 2009). Reflecting their natural lifestyle, orangutans in zoos also engage more in solitary play than the more gregarious great ape species, such as chimpanzees (Miller & Nadler, 1981). Despite lower interaction rates, orangutans show high social competence in the context-dependent use of play faces (Davila Ross et al., 2008; Waller et al., 2015) and play-solicitation gestures (Cartmill & Byrne, 2010; Fröhlich et al., 2021). Furthermore, like other great apes (Cordoni & Palagi, 2011), Sumatran orangutans (*Pongo abelii*) show different ontogenetic trajectories for social and solitary play (van Adrichem et al., 2006) in one very gregarious population (Mitra Setia et al., 2009; van Schaik, 1999).

Due to their semi-solitary lifestyle, dependent immature orangutans only have the opportunity for social play with individuals other than the mother when the mother associates with potential play partners. These occasions are rare, however, particularly in Bornean populations where females with unweaned offspring associate with others less than 10% of their active time (Kunz et al., 2021; Mitra Setia et al., 2009; van Noordwijk et al., 2012). The orangutans’ semi-solitariness is likely caused by limited food availability, as higher orangutan density, sociability and social tolerance in North-West Sumatran compared to North-East Sumatran and Bornean populations (Husson et al., 2009; Roth et al., 2020; Schuppli et al., 2017; van Schaik, 1999) align with higher forest productivity in these regions (Marshall et al., 2009; Wich et al., 2011). Yet, providing opportunities for offspring socialization was proposed to be the main benefit of female-female associations in Bornean orangutans (*Pongo pygmaeus*) (van Noordwijk et al., 2012). Within populations, mothers spend less time in association with each other during lean food periods which likely affects their offsprings’ play opportunities (Ashbury et al., 2022). Moreover, orangutan females are philopatric (Arora et al., 2012) and preferably associate with their maternal relatives in a Bornean population (van Noordwijk et al., 2012), which may imply an even more limited number of potential play partners.

We aimed to evaluate how play behavior relates to individual and socio-ecological factors in two populations of wild orangutans, one at Suaq, South Aceh, North-West Sumatra (*Pongo abelii*) and one at Tuanan, Central Kalimantan, Borneo (*P. pygmaeus wurmbii*). We assessed the three play types (social, solitary object and solitary locomotor play) separately, and evaluated variation with age, sex, study site, local fruit availability and social context. If the three different play types serve different developmental functions or reflect distinct levels of skill acquisition, we predicted distinct ontogenetic trajectories (age) (**P1**) and no compensation for a lack of social play by solitary play (**P2**). Moreover, if energetic costs limit play, we predicted its frequency would be positively correlated with local fruit availability (**P3**). If play behavior reflects adult skill repertoires, we predicted object play would be more prevalent in the Suaq population, where feeding techniques are more complex (Schuppli et al., 2017) and include frequent, sophisticated stick tool use (van Schaik et al., 1996), than at Tuanan, where tool use is absent (**P4**). Finally, if play behavior reflects adult sociability and serves socialization, we predicted that orangutans in the more sociable Sumatran population (Suaq) would show more social play than immature individuals of the less gregarious Bornean population (Tuanan) (**P5**).

In a semi-solitary species, variation in social play behavior may either be explained by association rates (i.e., opportunities to play), or interaction rates during these associations (i.e., the motivation to interact). Accordingly, if social play was mediated by association rates, we predicted that social play frequency would be higher for individuals from larger matrilines or with older siblings (**P6**) or in contexts (study sites) where association frequency is overall high (**P7**). If intrinsic motivation to play reflects higher adult sociability, we predicted the motivation to play to be higher at the more sociable population of Suaq compared to Tuanan (**P8**). Further, if play had a socializing function during ontogeny, we predicted a shift in play partners throughout immaturity (**P9**): from the mother and older sibling to additional association partners. Also following the socialization hypothesis, we predicted that mothers and/or siblings would not compensate for limited play (opportunities) with other associates (**P10**), but rather that association frequency with potential play partners would change over immatures' age (possibly regulated by the mother). Finally, if play interactions reflect adult sociability and social tolerance, we predicted more play with related individuals at Tuanan compared to Suaq, as a larger range of (unrelated) association partners may qualify as play partners in a setting of higher social tolerance (**P11**).

## Methods

### Study Site and Subjects

We conducted this study at two field sites in Indonesia: Suaq Balimbing (03°02’N; 97°25’E) in the Kluet region of Gunung Leuser National Park, South Aceh, Sumatra, and Tuanan (02°15’S; 114°44’E) in the Mawas Conservation Area, Central Kalimantan, Borneo. The study area in Suaq Balimbing consists of 500 ha primary, coastal peat-swamp forest. Suaq has the highest known orangutan density of 7 individuals per square kilometer (Singleton & van Schaik, 2001). The study area in Tuanan, Borneo, is situated in 750 ha of regenerating, formerly selectively logged peat-swamp forest. Tuanan has the highest reported orangutan density on Borneo with 4.5 individuals per square kilometer (van Schaik et al., 2005). Year-round, long-term studies on orangutan behavior are conducted at both field sites. For this study, we collected detailed behavioral data between June 2007 and March 2018 at Suaq and from July 2003 to June 2018 at Tuanan.

At both field sites, the studied orangutans are individually recognized. Based on demographic (van Schaik, 1999) and genetic data (Arora et al., 2012; Lenzi, 2014; Noordwijk et al., 2023), two major clusters of maternally related females (from here on referred to as *matrilines*) are known in Suaq. In Tuanan one central matriline of six females lives in the core of the study area and several mother–offspring units with fewer known close maternal relatives range more in the periphery of the study area (Table 1). At both sites, adult females have ranges that overlap with both closely related and non-related females (Arora et al., 2012; Ashbury et al., 2020; van Noordwijk et al., 2012). We accounted for the different matriline sizes in all analyses by adding a matriline size term, assigning immatures of females without known adult female relatives as *small* matrilines and all others to *large* matrilines (Table 1).

**Table 1:** Overview of study subjects and datasets analyzed in a study of play in Bornean (*Pongo pygmaeus wurmbii,* Tuanan, Central Kalimantan, Indonesia, 2003–2018, Tu) and Sumatran orangutans (*P. abelii,* Suaq Balimbing, South Aceh, Indonesia, 2007–2018, Su)

Site	ID	Sex	Birth	Matriline size^‡^	Sibling		Age range (y)	Number of full-day focal follows for	
					older	younger		ontogenetic play trajectory^†^	social play in dyadic associations^#^ *(mean* ± *SD association partners per day)*
Su	Lil	f	*mid 2001*	large	no	yes	6.6 - 9.9	8	0
Su	Chy	f	*early 2003*	large	NA	yes	8.2 - 8.8	0	3 (2.3 ± 0.6)
Su	Fre	m	*mid 2005*	large	yes	yes	2.48.8	14	12 (1.6 ± 0.7)
Su	Lo	m	Aug 2010	large	yes	yes	0.5 - 7.6	43	69 (2.5 ± 1.9)
Su	Cin	f	Apr 2012	large	yes	no	1.1 - 5.2	29	43 (3.3 ± 2.4)
Su	Fra	m	Aug 2012	large	yes	no	0.8 - 5.6	33	50 (2.2 ± 0.9)
Su	Si	m	Mar 2013	small	yes	no	0.6 - 0.9	0	12 (2.9 ± 1.6)
Su	Ren	m	July 2013	large	yes	no	0.7 - 0.8	10	11 (2.6 ± 1.6)
Su	Dal	f	Oct 2013	small	yes	no	2.7 - 2.7	0	2 (1.5 ± 0.7)
Su	Ed	f	Nov 2014	large	no	no	0.1 - 4.1	27	45 (2.7 ± 1.7)
Su	Am	m	early 2015	small	yes	no	0.1 - 2.7	0	2 (2.0 ± 1.4)
Su	Lut	m	Mar 2016	large	no	no	1.3 - 1.4	0	3 (3.0 ± 3.5)
Tu	Kon	f	*Jan 1999*	large	NA	yes	5.7 - 10	29	0
Tu	Mil	f	*mid 2001*	large	NA	yes	2.1 - 10.9	83	8 (2.1 ± 0.4)
Tu	Str	f	*mid 2002*	small	yes	yes	6.2 - 8.5	6	20 (2.4 ± 0.9)
Tu	Sus	f	*end 2002*	small	no	no	1.6 - 3.6	40	0
Tu	Jer	m	Jun 2003	large	yes	yes	0.1 - 8.6	84	13 (1.8 ± 1.2)
Tu	Der	m	Jul 2004	large	no	yes	1.0 - 8.1	12	6 (2.5 ± 0.8)
Tu	Jip	m	10.02.2006^ k^	large	no	yes	0.0 - 10.5	94	92 (2.3 ± 1.2)
Tu	Kin	m	Jan 2007	large	yes	yes	0.1 - 9.6	52	68 (2.5 ± 1.5)
Tu	Ips	f	Jun 2007	large	yes	yes	3.4 - 10.2	32	58 (2.3 ± 1.0)
Tu	Pet	m	Jun 2008	small	yes	yes	4.1 - 9.7	35	64 (2.3 ± 0.9)
Tu	Maw	f	15.07.2008^ k^	large	yes	yes	0.1 - 9.7	87	102 (2.0 ± 1.0)
Tu	Chr	m	mid 2008	small	NA	yes	5.0 - 9.3	16	23 (2.2 ± 0.6)
Tu	Son	m	28.03.2010^ k^	small	yes	no	0.4 - 7.1	49	62 (1.9 ± 1.0)
Tu	Dan	m	Jul 2010	large	yes	yes	0.2 - 7.5	39	68 (1.9 ± 1.0)
Tu	Joy	m	Jun 2011	large	yes	no	0.3 - 5.7	45	83 (1.4 ± 0.9)
Tu	Tuk	m	early 2012	small	yes	no	3.7 - 6.0	17	47 (2.0 ± 0.9)
Tu	Kah	f	Feb 2012	large	no	no	0.3 - 2.3	16	19 (1.8 ± 1.0)
Tu	Jan	f	Jun 2013	large	yes	yes	0.8 - 5.0	29	51 (1.7 ± 1.3)
Tu	Iya	m	Dec 2013	large	yes	no	0.3 - 3.4	17	31 (2.0 ± 0.9)
Tu	Ket	m	30.06.2014^ k^	large	yes	no	1.6 - 3.9	29	45 (1.4 ± 0.9)
Tu	Cha	m	Sep 2014	small	yes	no	1.8 - 3.6	11	17 (2.3 ± 0.8)
Tu	Mob	f	Nov 2014	large	yes	no	1.4 - 3.5	21	44 (1.6 ± 1.1)
Tu	Kil	m	Feb 2015	large	no	no	1.3 - 1.5	0	10 (1.1 ± 0.3)
Tu	Mer	m	Dec 2015	large	no	no	0.4 - 2.4	17	31 (1.5 ± 0.8)
Tu	Zak	f	early 2016	small	no	no	2.1	0	2 (2.0 ± 1.4)
Tu	Pin	f	15.05.2016^ k^	small	yes	no	1.1	0	2 (3 ± 0)
Tu	Dar	m	11.06.2016^ k^	large	yes	no	0.9- 1.9	13	13 (2.5 ± 0.7)
Total								1037	1231 (2576 associations)

**‡** from Arora et al., 2012; Lenzi, 2014; van Noordwijk et al., 2023

**†** only selected observers (researchers focusing on immature development)

# focal data between 2010 and 2018 from dependent and semi-dependent immatures included, all association partners counted here (own mother, other dependent, semi-dependent and independent immatures, adult females, and unflanged and flanged males)

^k^ known birth date

Birth months and years in italics indicate estimates for immatures present at the start of the study period

While we analyzed play trajectories using absolute age (Table 1), we defined three different age classes of immature individuals to categorize social play partners. *Dependent immatures* comprise all not yet weaned individuals. *Semi-dependent immatures* do not nurse anymore, but still spend most of their time in association with their mothers. *Independent immatures* range independently of their mothers. We refer to these three categories collectively as immatures, and to both semi-dependent and independent immatures as *weaned immatures*. We knew the sex of all immatures, but we estimated some ages to the nearest half year (ages of young infants that were born in recent years were estimated to the closest month) (Table 1). At Suaq, we followed 12 immatures (5 females and 7 males, born between 2001 and 2016) regularly. At Tuanan, we followed 27 immatures (11 females and 16 males, born between 1999 and 2016) regularly (Table 1).

### Behavioral Data

We used instantaneous behavioral sampling, following an established protocol (https://www.aim.uzh.ch/en/research/orangutannetwork/sfm.html) during full-day focal follows (mean 11.11 ± SD 1.10 focal hours per day), from the time when individuals left their morning nest to when they entered their evening nests. We recorded the focal individual's activity, visibility, and distance to other individuals at 2-min intervals. We defined three play categories as follows:

- *Solitary Object Play*: Manipulation of objects without an apparent immediate goal, often repetitive, e.g., swinging twigs, ripping off and discarding leaves, shaking, and manipulating sticks. We included *explorative* object manipulation (cf. Schuppli et al., 2016a, b) in this category.
- *Solitary Locomotor Play*: Repetitive movement of body parts or the whole body, like twirling up-side-down or swinging arms and/or legs. Solitary locomotor play can be in one spot, but also includes moving around with no obvious directed displacement.
- *Social Play*: Non-aggressive interaction between two or more individuals that does not serve any apparent, immediate purpose, like repetitive tagging, wrestling, and chasing. Social play includes body contact in most instances and is often accompanied by play faces or play vocalizations.

For each 2-min bout, we noted only one behavioral category. If the activity was a mix of categories at the 2-min scan, observers documented the behavior in a hierarchical order (social > solitary object > solitary locomotor play). Because the two solitary play types were often combined within a single play bout (mean 11.3 ± SD 6% of solitary play bouts is a combination, Fig. S1), we also analyzed overall solitary play frequency (Table S1). We labelled all individuals (including the own mother, younger or older siblings and any other individual) within a 50 m radius of the focal as *association partners*. Well-trained observers collected data, and passed an interobserver reliability test with an experienced observer with a Cohen's kappa of k ≥ 0.8 after an intense training phase. Several observers collected data at both study sites, further ensuring consistency in classification of play categories.

### Ecological Data

We calculated Fruit Availability Indices (FAI: percentage of trees with fruits) from monthly phenology surveys in strip plots with ~ 1600 (Tuanan) and ~ 1000 (Suaq) trees (Harrison et al., 2010; Vogel et al., 2015, 2017). Because fruit availability is generally higher at Suaq (range 3.35—17.4%, median 9.8; mean 9.91 ± 3.07, N = 86) than Tuanan (range 0.1—14.0%, median 3.69; mean 4.29 ± 2.65, N = 185 months), we z-transformed the values for each study site to assess effects of fruit availability within rather than between sites. We refer to the transformed index as zFAI or local fruit availability.

### Statistical Analysis

We used two main datasets for this study. First, we analyzed ontogenetic play trajectories and association frequency using longitudinal, 2-min ‘instantaneous’ scan data from full-day focal follows of immatures for whom at least 6 observation days were available over the study period (N = 1037 full-day focal follows; Table 1; Fig. S2). We included only data for dependent and semi-dependent immatures aged 11 years and younger, as males start to disperse around the age of 11 years (unpubl. data). Second, we used data on dyadic associations during full-day focal follows to analyze the occurrence of social play with specific partner classes (mother, sibling [older and younger] and associates [dependent, semi-dependent and independent immatures, and unflanged males]). We included data for dependent and semi-dependent immatures, who still ranged with their mother most of their active time, in these analyses (N = 2576 dyadic associations; Table 1).

We conducted data analysis using R version 4.2.1 (R Core Team, 2022). We performed Generalized Linear Mixed-effect Models (GLMMs) using the package ‘glmmTMB’ (Brooks et al., 2017). We checked models for heteroscedasticity and influential cases, also on the level of all random intercepts, using the package ‘DHARMa’ (Hartig & Lohse, 2021) and the model evaluation functions provided by the 'glmmTMB' package developers (https://glmmtmb.github.io/glmmTMB/articles/model_evaluation.html). If we detected influential cases, we reran analyses excluding them to determine whether we found the same patterns. We examined models assuming a Poisson error distribution for overdispersion (Bolker, 2021), and used negative binomial GLMMs instead if overdispersion appeared to be an issue. We generated all figures using ‘ggplot2’ (Wickham, 2016) and ‘ggeffects’ (Lüdecke, 2018, 2020) to illustrate model predictions. We conducted likelihood ratio tests to compare the full models (including all fixed effects) to their corresponding null model (including only offsets and random effects). We only report second order interaction terms when adding them further improved model fit based on likelihood ratio tests and lower AIC values. We report model estimates and standard errors, exponentiated estimates and 95% confidence intervals from the confint() function in the result tables. We used the alpha value of P < 0.05 as cut-off for significance.

#### Ontogenetic Play Trajectories (P1—P6)

First, we tested if the three play types follow different age trajectories using a negative binomial GLMM. We pooled daily 2-min scan counts of each play type in one analysis and evaluated if the second order interaction terms between play type and the linear and quadratic term of age explained a significant amount of variation in the occurrence of play. We included the total count of daily 2-min scans with known activity as an offset term using a log-link function (we report details of accounting for visibility constraints in the supplementary materials). To avoid pseudo-replication resulting from having the same individual during consecutive days in the analyses, we included the follow period (FP), i.e., the calendar year and calendar month of focal observation, nested in individual identity (ID) as random intercepts in all the models (~ 1|ID/FP). An individual follow period comprised a mean of 3 ± 2.3 full-day focal follows (range: 1–11; Fig. S2). Additionally, we included observer identity and observation day as a random intercept. Second, we evaluated the total count of daily 2-min scans spent in solitary object, solitary locomotor, or social play during full-day focal follows in three separate analyses (N = 1037). We assessed variation in daily play scan counts in relation to study site, age (linear and quadratic term), sex, and zFAI in negative binomial GLMMs. We additionally assessed if time devoted to social play varied with matriline size (large vs. small) or with the presence of an older sibling (no vs. yes). To assess if patterns differ between populations, we tested for second order interactions of study site with any other fixed factor in the analyses of all three play types. We included follow period nested in individual identity and observer identity as random intercepts, and daily known activity scans as offset term using a log-link function, as for the first analysis.

#### Play Opportunities – Association Frequency (P7)

We analyzed (i) the number of and (ii) cumulative association time (in 2-min scans) with potential play partners based on full-day focal follows with a subset of the data for which information on association duration and partners were available (N = 731 follow days). We labelled dependent, semi-dependent and independent immatures, and unflanged males as potential play partners, because we did not observe the other sex-age classes (flanged males and adult females) playing with immatures. We did not include the individual’s mother, with whom a dependent immature is always in association, or older siblings in these analyses. We included study site, age (linear and quadratic term), sex, matriline size, and zFAI as fixed factors and tested for any second order interaction terms of study site with the other fixed factors. As in the analyses of the play trajectories, we included follow period nested in focal identity as a random intercept. We included the total number of daily activity scans as an offset term using a log-link function when analyzing the duration of association.

#### Motivation to Play with Different Partners (P8—P11)

To investigate the variation in social play in detail, we first evaluated play occurrence during dyadic associations with different partner classes. We used a binomial GLMM to analyze the occurrence of social play (0/1) in a given dyadic association (N = 2576 dyadic associations [own mother: 1203; older sibling: 237; younger sibling: 241; other associate: 895]) with the total time in association (hours per day) as an offset term using a logit-link function. We included (i) focal identity nested in the follow period and (ii) the partner identity as crossed random intercept to avoid pseudo-replication. We added study site, age (linear and quadratic terms), sex and the partner class (mother, younger sibling, older sibling and associate) and zFAI as fixed effects. Further, we tested for second order interactions including either study site or age (either or both the linear and quadratic term) and all the other fixed factors to evaluate (i) potential site differences and (ii) age trajectory variation depending on other factors.

Second, we assessed the total count of observed social play scans in a given dyadic association per day. We evaluated play interactions with mothers, older siblings, and other associates in three different analyses. We set-up all GLMMs with a negative binomial error structure, included the total time in association as an offset term using a log-link function, and set the focal and partner identity as crossed random intercepts (~ 1|FocalID + 1|PartnerID). In all three analyses, we assessed social play bout variation in relation to study site, age (linear and quadratic term), sex, matriline size and zFAI as fixed factors. When evaluating play frequency with mothers, we also added the presence/absence of an older sibling as a fixed effect. In analyses of play with associates (non-sibling and non-mothers), we added the category of the partner (unweaned [dependent] immatures, weaned immatures [including semi-dependent and independent] and unflanged males) as fixed effect. In all analyses, we tested for second order interactions including either site, age or sex and any other factor.

## Ethical note

Behavioral data collection was non-invasive and exclusively observational. The data collection protocol for this study adheres to legal requirements of Indonesia and was approved by the Indonesian State Ministry for Research and Technology (RISTEK), the Directorate General of Natural Resources and Ecosystem Conservation- Ministry of Environment and Forestry of Indonesia (KSDAE-KLHK), the Ministry of Internal Affairs, Indonesia, the Nature Conservation Agency of Central Kalimantan (BKSDA) and Balai Besar Taman Nasional Gunung Leuser (BBTNGL).

### Data Availability

Data and source code for the analyses reported in the main text of this manuscript are available on the Harvard dataverse repository: https://doi.org/10.7910/DVN/F5FJJW.

### Conflict of Interest

The authors declare that they have no conflict of interest.

## Results

### Ontogenetic Trajectories of Play (P1—P6)

**Fig. 1:**
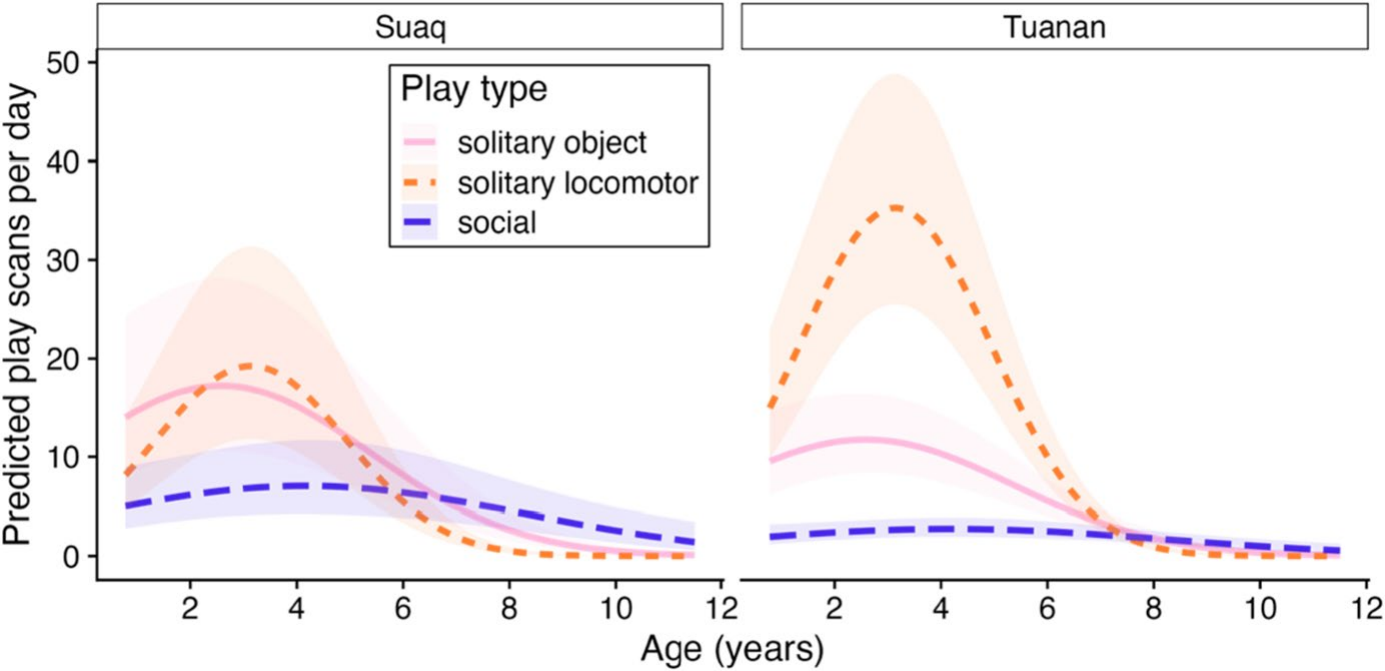
Play trajectories: Predicted number of daily 2-min scans spent in play behavior by immature orangutans by age (years) (x-axis), play type and study site (left: Sumatran orangutans (*P. abelii*) at Suaq Balimbing, South Aceh, Indonesia, 2007–2018; right: Bornean orangutans (*Pongo pygmaeus wurmbii*) at Tuanan, Central Kalimantan, Indonesia, 2003–2018). Lines indicate model predictions based on the full model for each study site and shaded areas illustrate 95% confidence intervals

The full model of daily play scans explained significantly more variation than the null model (Table 2a). Solitary object, solitary locomotor and social play showed distinct age trajectories (Fig. 1), as shown by the significant interaction terms between play type and both the linear and the quadratic term of age (Table 2a).

#### Solitary Object Play

The full model for solitary object play explained significantly more variation than the corresponding null model (Table 2b). The time immatures spent in solitary object play varied significantly with age (Fig. S3), but not with zFAI (Fig. S4), sex or the occurrence of social play on the same day (Table 2b). Solitary object play followed a quadratic age trajectory, increasing until around the age of 2 years and then decreasing towards the age of 6–8 years (Fig. 1). The age trajectories differed slightly but significantly between the two study sites, with a faster decline in solitary object play at Tuanan than at Suaq (Fig. 1), as shown by the significant interaction term between study site and the linear and quadratic age term (Table 2b). No other second order interaction term between study site and any of the fixed factors further enhanced the model fit.

#### Solitary Locomotor Play

The full model for solitary locomotor play explained significantly more variation than the corresponding null model (Table 2c). Solitary locomotor play showed a quadratic age trajectory which differed between the two study sites (Fig. S3), with a much higher peak in Tuanan than in Suaq (Table 2c; Fig. 1). Solitary locomotor play frequency did not vary significantly with local fruit availability (Fig. S4), sex or the occurrence of social play (Table 2c). No other interaction terms between study site and any of the fixed factors further improved the model fit.

#### Social Play

The full model explained significantly more variation in social play scans than the corresponding null model (Table 2d). Age trajectories in social play differed between the two study sites (Fig. S3). Social play followed a quadratic age trajectory at Suaq, the more sociable population, and peaked around the age of 4–6 years, whereas at Tuanan this peak was less pronounced, and social play frequency remained low(er) at Tuanan (Table 2d; Fig. 1). There was a significant interaction between zFAI and study site (Table 2d): Daily social play increased with increasing zFAI at Tuanan, whereas there was no such correlation at Suaq (Fig. S4), where absolute fruit availability is generally higher. Daily social play scans did not differ significantly between sexes, matriline size or if the immature had an older sibling (Table 2d).

**Table 2:** Solitary and social play frequency: Effects of individual, social and ecological variables on the daily 2-min play scan count per full-day focal follow by dependent and semi-dependent immature Bornean (*Pongo pygmaeus wurmbii* at Tuanan, Central Kalimantan, Indonesia, 2003–2018) and Sumatran (*P. abelii* at Suaq Balimbing, South Aceh, Indonesia, 2007–2018) orangutans, as obtained from negative binomial GLMMs. Sample sizes and likelihood ratio test model comparisons to the corresponding null model are shown below the response variables. Significant fixed effects (P < 0.05) are indicated in bold

Response	Variables	Exponentiated Coefficient	95% Confidence Interval	z	P
**a) Play scans** per full-day focal follow	Intercept	0.04	-3.375, -2.896	-	-
	ID (*N* = 31) / Follow period (N = 343)	*random intercept*			
	Observer (*N* = 49)	*random intercept*			
	Observation day (*N* = 1037)	*random intercept*			
	Total 2-min activity bouts	*offset (log-link)*			
	Play type				
	solitary locomotor vs. object play	0.53	-0.761, -0.504	-	-
	solitary locomotor vs. social play	0.24	-1.575, -1.254	-	-
	z Age	0.06	-3.175, -2.541	-	-
	z Age^2^	0.12	-2.430, -1.815	-	-
	**z Age: Play type**				
	z Age: (locomotor vs. object play)	4.08	1.101, 1.712	**9.020**	**< 0.001**
	z Age: (locomotor vs. social play)	12.11	2.180, 2.807	**15.579**	** < 0.001**
	**z Age** ^**2**^ **: Play type**				
	z Age^2^: (locomotor vs. object play)	3.91	1.073, 1.655	**9.186**	**< 0.001**
	z Age^2^: (locomotor vs. social play)	6.11	1.489, 2.131	**11.053**	**< 0.001**
	*N* = 3111 daily play type scans *χ*^*2*^_6,14_ = 1324, P < 0.0001, ΔAIC = 1308.0, R^2^_c_ = 0.72				
**b) Solitary object play scans** per full-day focal follow	Intercept	0.04	-3.874, -2.814	-	-
	ID (*N* = 31) / Follow period (N = 343)	*random intercept*			
	Observer (*N* = 49)	*random intercept*			
	Total 2-min activity scans	*offset (log-link)*			
	Site (Suaq vs. Tuanan)	0.44	-1.343, -0.290	-	-
	z Age	0.53	-1.102, -0.168	-	-
	z Age^2^	0.57	-1.105, -0.033	-	-
	**Site: z Age**	0.18	-2.250, -1.125	**-5.881**	** < 0.001**
	**Site: z Age** ^**2**^	0.42	-1.482, -0.232	**-2.687**	**0.007**
	Sex (female vs. male)	0.86	-0.444, 0.144	-0.998	0.318
	z FAI	1.04	-0.072, 0.143	0.647	0.518
	Social play (0/1)	1.00	-0.129, 0.122	-0.052	0.959
	N = 1037 full-day focal follow days [Suaq: N = 164; Tuanan: N = 873] *χ*^*2*^_5,13_ = 221.16, P < 0.0001, ΔAIC = 205.16				
**c) Solitary locomotor play scans** per full-day focal follow	Intercept	0.02	-4.786, -3.201	-	-
	ID (*N* = 31) / Follow period (*N* = 343)	*random intercept*			
	Observer (*N* = 49)	*random intercept*			
	Total 2-min activity scans	*offset (log-link)*			
	Site (Suaq vs. Tuanan)	1.82	-0.186, 1.385	-	-
	z Age	0.25	-2.146, -0.606	-	-
	z Age^2^	0.44	-1.639, 0.011	-	-
	**Site: z Age**	0.09	-3.254, -1.458	**-5.143**	** < 0.001**
	**Site: z Age** ^**2**^	0.13	-2.975, -1.094	**-4.239**	** < 0.001**
	Sex (female vs. male)	0.86	-0.659, 0.346	-0.610	0.542
	z FAI	1.08	-0.027, 0.186	1.467	0.142
	Social play (0/1)	1.10	-0.005, 0.187	1.852	0.064
	*N* = 1037 full-day focal follow days [Suaq: *N* = 164; Tuanan: *N* = 873] *χ*^*2*^_5,13_ = 300.82, P < 0.0001, ΔAIC = 284.82				
**d) Social play scans** per full-day focal follow	Intercept	0.02	-4.524, -2.997	-	-
	ID (*N* = 31) / Follow period (N = 343)	*random intercept*			
	Observer (*N* = 49)	*random intercept*			
	Total 2-min activity scans	*offset (log-link)*			
	Site (Suaq vs. Tuanan)	0.34	-1.780, -0.405	-	-
	**z Age**	0.69	-0.613, -0.125	**-2.968**	**0.003**
	z Age^2^	0.40	-1.596, -0.249	-	-
	**Site: z Age** ^**2**^	2.15	0.042, 1.486	**2.074**	**0.038**
	Sex (female vs. male)	1.26	-0.182, 0.640	1.093	0.274
	Matriline (large vs. small)	0.64	-0.978, 0.086	-1.645	0.100
	Older sibling (no vs. yes)	1.31	-0.193, 0.726	1.139	0.255
	z FAI	0.81	-0.530, 0.099	-	-
	**Site: z FAI**	1.63	0.129, 0.844	**2.664**	**0.008**
	*N* = 1037 full-day focal follow days [Suaq: *N* = 164; Tuanan: *N* = 873] *χ*^*2*^_5,14_ = 39.00, P < 0.0001, ΔAIC = 21.00				

### Play Opportunities—Association Frequency (P7)

As predicted, both the mean number and the mean cumulative time spent in associations with potential play partners, including dependent, semi-dependent und independent immatures, and unflanged males, were higher at Suaq (1.25 ± SD 1.44 potential play partners per full-day focal follow and 4.45 ± SD 7.98 cumulative association hours) than at Tuanan (0.78 ± SD 0.84 partners and 1.22 ± SD 2.92 cumulative hours). While both number and duration of associations with potential play partners increased with local fruit availability at Tuanan, they remained constant or even decreased at Suaq, as the interaction between site and zFAI shows (Table 3; Fig. S5). The overall number of association partners did not vary with age or sex of the immature (Table 3). However, in separate analyses of association partner types, the number of dependent immatures and unflanged males increased and the number of weaned immatures decreased with the age of immatures (Table S2; Fig. S6).

**Table 3:** Effects of individual, social and ecological variables on the number of play partners and cumulative duration of association with them (includes dependent, semi-dependent and independent immatures, and unflanged males) during full-day focal follows on Bornean (*Pongo pygmaeus wurmbii* at Tuanan, Central Kalimantan, Indonesia, 2003–2018) and Sumatran orangutans (*P. abelii* at Suaq Balimbing, South Aceh, Indonesia, 2007–2018). Sample size, model error distribution and likelihood ratio test model comparisons to the null models are indicated below the response variables. Significant fixed effects (P < 0.025, with Bonferroni adjustment) are indicated in bold

Reponse	Variables	Exponentiated Coefficient	95% Confidence Interval	z	P
**a) Number of associations with potential play partners**	Intercept	1.16	-0.141, 0.435	-	-
	ID (*N* = 29) / Follow period (*N* = 261)	*random intercept*			
	Site (Suaq vs. Tuanan)	0.60	-0.805, -0.207	-	-
	z FAI	0.81	-0.425, 0.001	-	-
	**Site: z FAI**	1.46	0.121, 0.630	**2.893**	**0.004**
	z Age	1.16	-0.010, 0.299	1.836	0.066
	Sex (female vs. male)	1.00	-0.239, 0.234	-0.022	0.982
	Matriline (large vs. small)	1.21	-0.120, 0.493	1.194	0.232
	*N* = 731 full-days focal follows χ^2^_3,10_ = 20.04, P = 0.003, ΔAIC = 8.02 (Poisson GLMM)				
**b) Total 2-min scans in association with potential play partners**	Intercept	0.27	-1.928, -0.681	-	-
	ID (*N* = 29) / Follow period (*N* = 260)	*random intercept*			
	Total active time	*offset (log-link)*			
	Site (Suaq vs. Tuanan)	0.29	-1.852, -0.599	-	-
	z FAI	0.79	-0.603, 0.129	-	-
	**Site: z FAI**	1.79	**0.126, 1.036**	**2.504**	**0.012**
	z Age	1.00	-0.313, 0.306	-0.021	0.983
	Sex (female vs. male)	0.90	-0.643, 0.436	-0.377	0.706
	Matriline (large vs. small)	1.11	-0.558, 0.768	0.311	0.756
	*N* = 728 full-days focal follows χ^2^_4,10_ = 20.14, P = 0.003, ΔAIC = 8.14 (negative binomial GLMM)				

### Motivation to Play with Different Partners (P8—P11)

#### Probability of Play During Dyadic Association

**Fig. 2:**
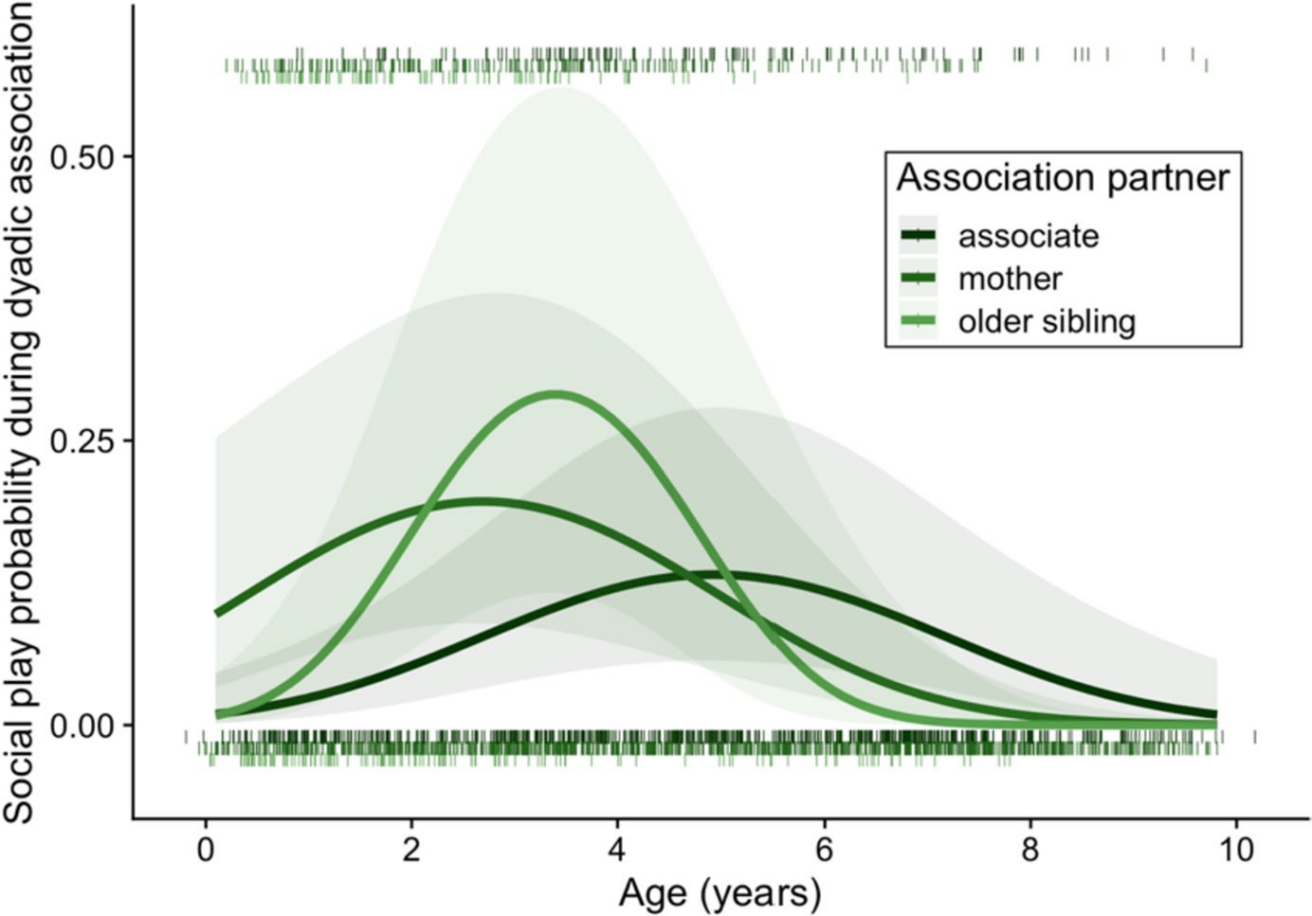
Probability of social play (0/1) during associations with different partners (associates, the immatures’ own mother and their older sibling) by the age of immature (years) in Bornean (*Pongo pygmaeus wurmbii* at Tuanan, Central Kalimantan, Indonesia, 2003–2018) and Sumatran orangutans (*P. abelii* at Suaq Balimbing, South Aceh, Indonesia, 2007–2018). Lines indicate the predicted probability of social play during a given dyadic association. 'Rugs' (i.e., the short vertical dashes at the bottom and top of the panel) indicate the distribution of associations (bottom: association, but no play observed; top: association and play observed), and shaded areas the 95% confidence intervals

The full model explained significantly more variation in social play occurrence during associations than the corresponding null model (Table 4). During an association, the probability of social play between the dyadic association partners varied with (i) partner type dependent on immatures' age (quadratic effect) (Fig. 2), (ii) sex, and (iii) study site depending on local fruit availability (Table 4). First, the probability of social play in an association with the older sibling peaked at an earlier age and decreased sooner than with other partners. Moreover, the probability of social play with the individual’s mother decreased at a younger age than with other (non-sibling) associates (Table 4). Second, male immatures were more likely to engage in social play than female immatures in a given dyadic association (Table 4). The second order interaction between sex and either (i) study site, (ii) partner type, or (iii) age did not further improve the model fit. Finally, the probability of social play in a dyadic association increased with increasing zFAI at Tuanan, but did not vary with zFAI at Suaq (Table 4).

**Table 4:** Effects of individual, social and ecological variables on the occurrence of social play in a given dyadic association, derived using a GLMM with binomial error structure** (**N = 2576 dyadic associations during full-day focal follows of immature focal individuals [Bornean (*Pongo pygmaeus wurmbii* at Tuanan, Central Kalimantan, Indonesia, 2003–2018) and Sumatran orangutans (*P. abelii* at Suaq Balimbing, South Aceh, Indonesia, 2007–2018)]). Comparisons to the null model based on a likelihood ratio test are indicated below the response variables. Significant fixed effects (P < 0.05) are indicated in bold

Response	Variables	Exponentiated Coefficient	95% Confidence Interval	z	P
**Probability of social play during dyadic association**	Intercept	1.67	-0.416, 1.443	1.083	0.279
	Focal ID (*N* = 36) / Follow period (*N* = 356)	*random intercept*			
	Partner ID (*N* = 213)	*random intercept*			
	Total time in association (h)	*offset term (logit-link)*			
	Site (Suaq vs. Tuanan)	0.93	-0.919, 0.778	-	-
	z Age	0.52	-1.406, 0.089	-	-
	z Age^2^	0.22	-2.411, -0.657	-	-
	**Sex (female vs. male)**	1.97	**0.145, 1.214**	**2.493**	**0.013**
	Matriline size (large vs. small)	0.60	-1.319, 0.307	-1.221	0.222
	Partner				
	associate vs. mother	0.57	-1.305, 0.170	-	-
	associate vs. older sibling	0.45	-2.303, 0.713	-	-
	associate vs. younger sibling	0.05	-6.400, 0.428	-	-
	**z Age: Partner**				
	z Age: (associate vs. mother)	0.14	**-2.993, -0.914**	**-3.684**	** < 0.001**
	z Age: (associate vs. older sibling)	0.01	**-8.842, -1.380**	**-2.685**	**0.007**
	z Age: (associate vs. younger sibling)	0.49	-11.626, 10.189	-0.129	0.897
	**z Age**^**2**^**: Partner**				
	z Age^2^: (associate vs. mother)	0.97	-1.087, 1.029	-0.054	0.957
	z Age^2^: (associate vs. older sibling)	0.05	**-5.473, -0.474**	**-2.332**	**0.020**
	z Age^2^: (associate vs. younger sibling)	152.60	-3.517, 13.572	1.153	0.249
	z FAI	0.62	-0.882, -0.061	-	-
	**Site: z FAI**	2.50	**0.438, 1.392**	**3.757**	** < 0.001**
	χ^2^_4,20_ = 114.32, P < 0.0001, ΔAIC = 82.3

#### Play Frequency with Own Mother

We observed immature focal individuals playing with their mother on 17.3 ± SD 37.9% (Suaq: 21.6 ± SD 41.2%; Tuanan: 16.3 ± SD 36.9%) of full-day focal follows and in a mean of 0.75 ± SD 2.51 (Suaq: 1.01 ± SD 2.62; Tuanan 0.68 ± SD 2.47) daily 2-min scans. The age trajectory of total daily play scans with the mother followed a quadratic function (Table 5a). While social play with their own mother was recorded for only a few scans per day at both sites (on average), the frequency increased somewhat with increasing zFAI at Tuanan, but remained constant with zFAI at Suaq, as indicated by the significant interaction term (Table 5a; Fig. S8). Social play with the own mother did not vary with sex or study site.

**Table 5:** Effects of individual, social and ecological variables on the count of 2-min social play scans per full-day focal follows with a) the mother, b) the older sibling, and c) association partners (unweaned and weaned immatures, and unflanged males) in Bornean (*Pongo pygmaeus wurmbii* at Tuanan, Central Kalimantan, Indonesia, 2003–2018) and Sumatran (*P. abelii* at Suaq Balimbing, South Aceh, Indonesia, 2007–2018) orangutans. Comparisons to the null models (fixed effect = 1, offset and random intercepts) based on likelihood ratio tests and the sample size (dyadic associations with the partner category on full-day focal follows) are indicated below the response variable. Significant fixed effects (P < 0.05) are indicated in bold

Response	Variables	Exponentiated Coefficient	95% Confidence Interval	z	P
**a) Social play scans with mother**	Intercept	0.63	-2.436, 1.525	-	-
	Focal ID (N = 36) / Follow period (N = 347)	*random intercept*			
	Mother ID (N = 25)	*random intercept*			
	Total time in association (h)	*offset term (log-link)*			
	Site (Suaq vs. Tuanan)	0.98	-0.669, 0.634	-	-
	zFAI	0.63	-0.857, -0.053	-	-
	**Site (Suaq vs. Tuanan): zFAI**	2.57	**0.476, 1.415**	**3.946**	** < 0.001**
	**z Age**	5.79	**0.280, 3.234**	**2.332**	**0.020**
	**z Age** ^**2**^	0.90	**-0.159, -0.054**	**-3.995**	** < 0.001**
	Sex (female vs. male)	1.55	-0.142, 1.017	1.479	0.139
	Matriline size (large vs. small)	1.01	-0.711, 0.731	0.027	0.978
	Older sibling (no vs. yes)	0.62	-1.093, 0.152	-1.481	0.139
	N = 1202 dyadic associations χ^2^_5,13_ = 75.58, P < 0.0001, ΔAIC = 25.09, R^2^_c_ = 0.30				
**b) Social play scans with older sibling**	Intercept	1778	4.346, 10.621	-	-
	Focal ID (N = 18) †	*random intercept*			
	Older sibling ID (N = 24)	*random intercept*			
	Total time in association (h)	*offset term (log-link)*			
	**Site (Suaq vs. Tuanan)**	0.42	**-1.486, -0.233**	**-2.690**	** < 0.001**
	zFAI	1.15	-0.078, 0.359	1.260	0.208
	**z Age**	487.7	**3.694, 8.686**	**4.861**	** < 0.001**
	**z Age** ^**2**^	0.78	**-0.351, -0.136**	**-4.446**	** < 0.001**
	Focal sex (female vs. male)	0.59	-1.221, 0.150	-1.531	0.126
	Sibling sex (female vs. male)	1.36	-0.393, 1.008	0.861	0.389
	Matriline size (large vs. small)	1.37	-0.456, 1.087	0.801	0.423
	N = 237 dyadic associations χ^2^_4, 11_ = 29.06, P < 0.0001, ΔAIC = 15.06, R^2^_c_ = 0.20				
**c) Social play scans with associates‡**	Intercept	1.29	-1.030, 1.539	-	-
	Focal ID (N = 35) / Follow period (N = 198)	*random intercept*			
	Partner ID (N = 138)	*random intercept*			
	Total time in association (h)	*offset term (log-link)*			
	Site (Suaq vs. Tuanan)	0.73	-1.305, 0.686	-	-
	Associate maternally related (no vs. yes)	0.53	-1.604, 0.344	-	-
	**Site: Associate maternally related**	5.30	0.404, 2.932	**2.586**	**0.010**
	z Age	0.54	-1.245, 0.029	-1.872	0.061
	**z Age** ^**2**^	0.36	-1.729, -0.288	**-2.744**	**0.006**
	Focal sex (female vs. male)	0.87	-1.036, 0.759	-	-
	Partner class^**‡**^				
	unweaned vs. weaned immature	0.19	-3.077, -0.254	-	-
	unweaned vs. unflanged male	0.02	-6.233, -1.824	-	-
	**Focal sex: Partner class** ^**‡**^				
	Sex: Partner (unweaned vs. weaned)	5.59	0.199, 3.242	**2.216**	**0.027**
	Sex: Partner (unweaned vs. unflanged)	29.14	1.100, 5.645	**2.908**	**0.004**
	z FAI	1.05	-0.204, 0.295	0.358	0.720
	Matriline size (large vs. small)	0.54	-1.673, 0.439	-1.146	0.252
	Older sibling (no vs. yes)	1.15	-0.771, 1.052	0.302	0.762
	N = 577 dyadic associations χ^2^_5, 18_ = 61.32, P < 0.0001, ΔAIC = 35.32, R^2^_c_ = 0.28				

† adding FP led to singularity issues

^**‡**^ weaned immatures includes both weaned, semi-dependent and independent immatures.

#### Play Frequency with Older Sibling

When they were in association, immature focal individuals played with their older siblings during 56.9 ± 49.9% (mean ± SD) of full-day focal follows at Suaq, and 33.7 ± 47.4% at Tuanan. Immature focal individuals spent more 2-min scans in social play per time in association with their older siblings at Suaq (4.0 ± SD 6.8) than at Tuanan (3.1 ± SD 7.1) (Table 5b). Daily play frequency (2-min scans) with older siblings followed a quadractic age trajectory but did not vary significantly with the sex of either the focal individual or the older sibling or with zFAI (Table 5b).

#### Play Frequency with Associates

**Fig. 3:**
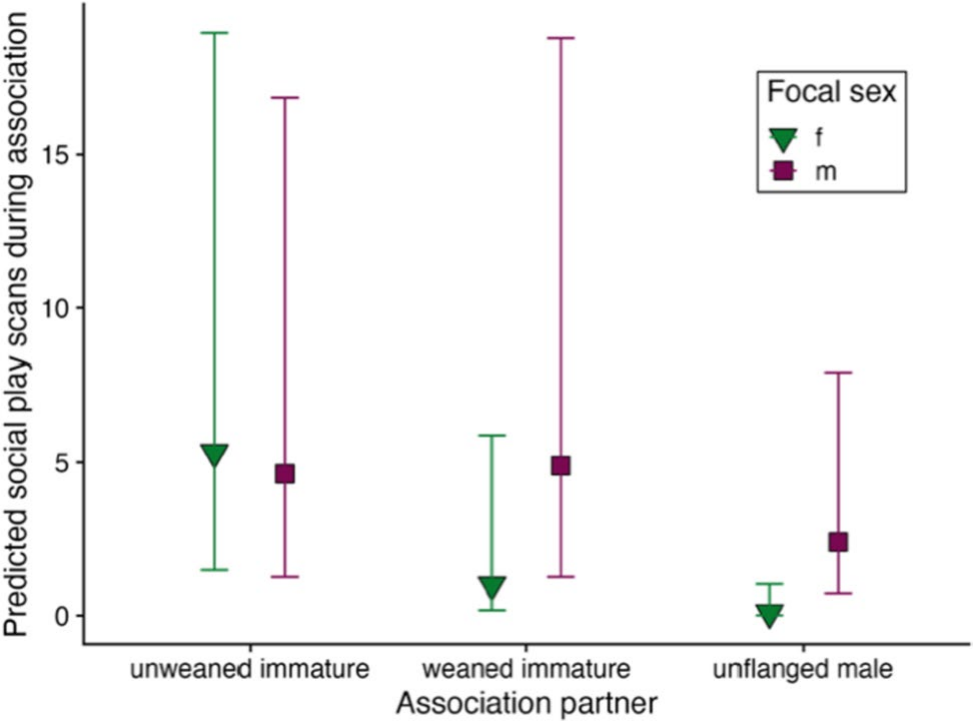
Predicted number of 2-min social play scans during association with different partners by the sex of immature focal individuals in Bornean (*Pongo pygmaeus wurmbii* at Tuanan, Central Kalimantan, Indonesia, 2003–2018) and Sumatran orangutans (*P. abelii at* Suaq Balimbing, South Aceh, Indonesia, 2007–2018). Symbols indicate model predictions and error bars show 95% confidence intervals obtained from the model (Table 5c)

The amount of social play with other associates, including dependent, semi-dependent, and independent immatures, and unflanged males, did not vary significantly with zFAI, study site, matriline size or if the focal had an older sibling, but did vary significantly as (i) a quadratic function of age, (ii) an interaction of focal sex and partner class, and (iii) an interaction of study site and if play partners were maternal relatives (Table 5c). First, male and female immatures did not differ significantly in their time playing with dependent immatures (male focal: 10.0 ± SD 24.4 scans; female focal: 14.8 ± SD 25.3 scans), but male immatures spent relatively more time in social play with weaned immatures (male focal: 5.4 ± SD 10.5 scans; female focal: 2.5 ± SD 8.8 scans) and unflanged males (male focal: 1.6 ± SD 5.5 scans; female focal: 0.1 ± SD 0.5), compared to female immatures (Fig. 3; Fig. S7 for distribution of raw data). Second, immatures' play frequency was higher with maternally related association partners than unrelated association partners at Tuanan, but not at Suaq, where play frequency did not depend on maternal relatedness (Fig. S9).

## Discussion

Play behavior in orangutans varies with age, sex, local fruit availability and socio-ecological context. As predicted, our results showed that solitary object, solitary locomotor and social play follow different age trajectories (P1), whereas we could not find any evidence for compensation effects between solitary and social play (P2). While we did not account for the combination of different play types, particularly of solitary play, our results may reflect developmental constraints on the expression of play or indicate that different play types serve different developmental functions. As predicted, fewer opportunities (P7) appear to cause the lower social play frequency at the less sociable population of Tuanan compared to Suaq (P5). Supporting our predictions and the socialization hypothesis, play partners changed during ontogeny (P9), and mothers did not compensate for the limited number of play opportunities with other play partners (P10). While solitary play was not affected by local fruit availability, social play decreased in all dyads at Tuanan at lower fruit availabilities (P3). Altogether, our findings support hypotheses that play behavior reflects adult behavioral repertoire and sociability, while being constrained by energy.

### Solitary Locomotor and Solitary Object Play Trajectories

Solitary play might be linked to skill acquisition during the first few years of life: Both solitary object and solitary locomotor play were most frequent during the first 4 years of life, and ceased almost completely around the age of weaning at 6 to 7 years. First, the drop in solitary locomotor play coincides with the start of consistent independent travel when the offspring is rarely carried by the mother during travel (Chappell et al., 2015; van Noordwijk et al., 2009). The trajectory of solitary locomotor play differed between study sites, with a more pronounced peak at Tuanan than at Suaq. We did not predict a site difference in solitary locomotor play, but it may be related to the different forest structure at the two sites, which may require different adult locomotor skill sets (Chappell et al., 2015; Manduell et al., 2012). The trees at Suaq tend to be large and thus the canopy is more 'rugged' than at Tuanan, where many small and still flexible trees fill most gaps. Future studies comparing locomotor play across study populations with varying forest structures are needed to further elucidate if locomotor play is more frequent in habitats with more challenging forest structures and may reflect increased need for motor skill training.

Second, solitary object play remained prevalent at older ages at Suaq (P4), where complex feeding techniques, including habitual, sophisticated (stick) tool use, are regularly observed (Schuppli et al., 2017; van Schaik et al., 1996), unlike at Tuanan where feeding techniques appear less complex and tool use is absent. However, the difference in solitary object play was modest. Moreover, immatures become proficient tool users well after the drop in solitary object play, around the age of 8 years at the earliest (Meulman et al., 2013). Solitary object play may thus be a first step in manipulation skills and understanding the physical concepts needed for tool use, combined with social learning (Schuppli et al., 2016a, b; sensu van Schaik & Burkart, 2011), and more fine-tuned practice in the form of exploratory activities (Schuppli, van Cauwenberghe et al., 2021b). More sophisticated adult behavior may further be foreshadowed by increasing complexity, particular manipulation, or specific types of objects during play rather than by absolute rates of play (Schuppli, van Cauwenberghe et al., 2021b). In the genus *Pan,* chimpanzees in populations with frequent stick tool use showed a higher use of detached objects with increasing age, and more varied object manipulation than bonobos, where such stick tool use is absent (Koops et al., 2015a, b; Koops et al., 2015a, b; Myowa-Yamakoshi & Yamakoshi, 2011)*.* Object manipulation quality also foreshadowed particular skill sets in a chimpanzee population without habitual tool use (Lamon et al., 2018).

Future studies will show whether the nature of object play, such as the objects used, or distinct manipulative actions performed, rather than frequencies per se further reflect the differences between populations with or without complex feeding techniques in orangutans. Object play may generally serve motor skill acquisition (e.g., geladas [*Theropithecus gelada*]: Cangiano & Palagi, 2020). The slightly extended period of solitary object play at Suaq compared to Tuanan might be linked to slower developmental trajectories overall, as overall slower development and a slower acquisition of feeding skills has been suggested for Sumatran compared to Bornean orangutans (Schuppli, et al., 2016a, b; van Noordwijk et al., 2009). For now, we conclude that there is only a minor predisposition or motivational difference for increased overall play frequencies with objects in early life phases between populations with different levels of feeding technique complexity in orangutans.

### Social Play

As predicted, social play frequency reflected population sociability (P5). At the less sociable population of Tuanan, social play remained constant throughout immaturity and decreased towards adolescence, with large variation between days. The time spent on social play was generally higher and peaked around the age of 4 to 6 years at Suaq, the more sociable population. The higher individual social play frequency at Suaq compared to Tuanan, however, is the direct result of the increased association frequency, and hence more play opportunities (P7), rather than the immatures' intrinsic motivation to play (P8). At Tuanan, immatures (i) were as likely to play and (ii) spent as much time in social play within a given association as at Suaq (cf. Fröhlich et al., 2020). The only exceptions were older siblings with whom play frequency was generally higher at Suaq than at Tuanan, which can likely be explained by the energetic costs of play. Indeed, the lower association frequencies and hence fewer play opportunities at Tuanan compared to Suaq may increase the immatures’ motivation to play once they get the chance, and their mother associates with potential play partners. Thus, we may see a compensation effect at Tuanan, which is not necessary at Suaq due to the higher availability of play partners. Associations also lasted longer at Suaq and there may have been a saturation effect on motivation or energy to play. In sum, these findings support the basic rule ‘play when you get the opportunity’ unless limited by energy. However, we cannot infer the directionality of the relationship between amount of social play and adult social competence posited by the social skills hypothesis (Baldwin & Baldwin, 1974) or the socialization hypothesis (Fagen, 1981).

#### No Compensation for Limited Play Opportunities by the Mother

The availability of play partners is a limiting factor for social play in orangutans given their semi-solitary lifestyle. Mothers do not appear to make up for the limited play opportunities for their offspring (P10). First, when associations with other partners were rare, during extremely low fruit availability, play frequency with mothers was also low. Second, neither mothers nor siblings in small matrilines, with fewer observed social play opportunities (van Noordwijk et al., 2012), played more with their offspring or younger sibling, respectively. Third, play partner identity transitioned from the mother to other individuals with increasing age supporting our prediction (P9). From the age of 4 years onwards, immatures hardly played with their own mothers. Our finding of changing play partners during ontogeny supports the socialization hypothesis and is in accordance with reports on released orangutans (Mendonça et al., 2017; Rijksen, 1978), chimpanzees (Cordoni & Palagi, 2011), bottlenose dolphins *(Tursiops truncatus*: Mackey et al., 2014) and humans (Pellegrini, 2009). It remains to be investigated whether play quality in wild orangutans also changes with age, or play partner and context, as for example in chimpanzees (Cordoni & Palagi, 2011; Flack et al., 2004).

#### Do Mother Drive Socialization in their Offspring

Social play facilitates the acquisition of social competence and rank (e.g., chimpanzees: Shimada & Sueur, 2014), but social competence is also required to maintain social play bouts (e.g., van Leeuwen et al., 2013). In species with high fission–fusion dynamics, such as orangutans or chimpanzees, associations tend to be costly for nursing mothers (Kunz et al., 2021), which limits the access their dependent offspring have to potential playmates. Active play partner choice by immatures may therefore rarely be feasible, unless mothers drive socialization via selective associations (Murray et al., 2014; Williams et al., 2002). We found only limited evidence for this: while overall association frequency did not vary with the age or sex of the immature, association frequency with different partner types varied with the age of immatures, which might indicate some selection of association (and play) partners by the mothers. Further, mothers’ preference for associating with maternal relatives has been suggested to lead to more social play opportunities for immatures in larger matrilines (van Noordwijk et al., 2012). We did not observe overall differences in social play frequency in immatures from larger versus smaller matrilines but we did find a difference in social play frequency with maternally related associates compared to unrelated associates at Tuanan, although not at Suaq. This finding indicates that maternal relatives are indeed preferred play partners, at least in one less sociable population, where social tolerance may be a limiting factor for play to occur.

#### Sex Differences in Social Play Probability

While overall social play frequency did not differ between male and female immatures, immature male orangutans were more likely to engage in social play during dyadic associations and showed higher play frequencies with other weaned immatures and unflanged males than immature females did. Similar sex differences in orangutan social play behavior have been found in rehabilitation centers (Descovich et al., 2011), and they match sex differences in adult orangutan skill sets and their acquisition (Ehmann et al., 2021; Schuppli et al., 2021a, b). Immature male orangutans eventually leave their natal home range (Arora et al., 2012), and show relatively high levels of sociability post-dispersal and even regular playful interactions (Fox, 2002; Galdikas, 1985; Mörchen et al., 2023, n.d; Utami Atmoko et al., 2009). Whether social play serves social skill acquisition, to practice fighting skills or to reduce tension (e.g., Verreaux’s sifaka [*Propithecus verreauxi*]: Antonacci et al., 2010; Palagi, 2018) in (male) orangutans remains to be tested with more detailed data on social play quality. Unfortunately, longitudinal studies evaluating play behavior in relation to social and skill competence in adolescent and unflanged male orangutans are hardly feasible in the wild due to their natal dispersal (Arora et al., 2012). Immature female orangutans, in contrast, appeared to play slightly more with dependent immatures than with weaned immatures and unflanged males. Although our data set is one of the largest available on wild orangutan populations, we are still limited in accounting for potential confounding variables such as demography, dyad composition, and age and sex differences between play partners due to orangutans' slow life history (van Noordwijk et al., 2018) and semi-solitary lifestyle (van Schaik, 1999), which make associations and social interactions rare. Thus, the sex differences we found should be treated with caution, although they are in line with earlier studies of orangutans and other more gregarious great ape species (chimpanzees: Kahlenberg & Wrangham, 2010; Koops et al., 2015a, b; Lonsdorf et al., 2014a, b; Pusey, 1990; gorillas: Maestripieri & Ross, 2004).

#### Energetic Constraints to Play

Play is presumably only feasible when animals have surplus energy (Berghänel et al., 2015; Held & Špinka, 2011; Palagi, 2018; Spencer, 1872). Accordingly, social play frequency increased with increasing local fruit availability at Tuanan, although this increase was a few minutes per day at most, whereas it did not vary with local fruit availability at Suaq. Although these differences may be in part attributed to association patterns, and hence, decreased opportunities to play at lower local fruit availability at Tuanan, the motivation, or rather energy, to play appears to decrease with decreasing local fruit availability at Tuanan. Moreover, social play frequency with the mother also decreased at lower fruit abundance at Tuanan. While this effect was small and social play with the mother is usually very gentle, it suggests that mothers might be particularly reluctant to play when only low-value nutrition is available. This pattern fits the notion that mothers oversee social play opportunities for their dependents for which they have to provide the additional energy via milk and transport (carrying) (van Noordwijk et al., 2013). Importantly, social play did not cease completely even during extended periods of very low fruit availabilities (Ashbury et al., 2022). This finding suggests that the benefits of low but persistent rates of social play outweigh the costs of social play even during lean periods.

Contra our prediction (P3), solitary play frequency did not vary with local fruit availability and thus, immatures appear to be cost-insensitive. Mothers have less control over solitary play despite acting as an energy buffer for their offspring. Therefore, it is not surprising that solitary play ceases by the time of weaning. The energy expenditure for solitary play may thus represent a hidden mother–offspring conflict (sensu Trivers, 1974).

## Conclusion

We show that solitary object, solitary locomotor, and social play follow different ontogenetic trajectories in orangutans. Play seems crucial during the ontogeny of immature orangutans, as the intrinsic motivation to play appears largely independent of the socio-ecological context. However, social play frequency is shaped by variation in sociability and ecology across two populations. Assessing the potential functions of play and specifically, whether individual play experience translates into adult competence, survival, and reproductive success, will only be feasible through extended longitudinal studies, and will be easier in the philopatric females. Our study supports growing evidence that both solitary and social play behavior changes during ontogeny and its variation reflects adult behavioral repertoires. While play behavior may be limited by energy and skill development to some extent, the intrinsic motivation of immature individuals to play suggests that it has developmental functions.

### Supplementary Information

Below is the link to the electronic supplementary material.

Supplementary file1 (DOCX 15863 KB)

## Declarations

### Inclusion and Diversity Statement

The author list includes contributors from the location where the research was conducted, who participated in study conception, data collection, interpretation of the findings, and long-term project administration.
