# Supplementary material for: Play Behavior Varies with Age, Sex, and Socioecological Context in Wild, Immature Orangutans (Pongo spp.)
Source: Int J Primatol. 2024 Jan 20;45(4):739–73. doi: 10.1007/s10764-023-00414-2 (PMC11339113; doi:10.1007/s10764-023-00414-2)
Supplement: Supplementary file 1 — Supplementary file1 (DOCX 15863 KB) [file 10764_2023_414_MOESM1_ESM.docx]

**Play behavior varies with age, sex and socio-ecological context in wild, immature orangutans (*Pongo* sp.)**

# Methods

## *Accounting for visibility constraints*

Because orangutans are almost exclusively arboreal (Ashbury et al. 2015), the canopy may obstruct visibility, especially for very young, small individuals. Because a specific visibility assessment was only introduced into the established protocol since mid 2012, bouts scored as ‘unknown activity’ were taken as a proxy for visibility bias in the long-term data sets. Bouts with unknown activity and 0 visibility scores correlated strongly (1.06 ± 0.12, t=9.23, P < 0.0001, F_1,71_=84.98, P<0.0001, N=73), and thus, present a good proxy for the visibility bias.

*Combination of solitary object and solitary locomotor play*

In the standardized data collection protocol, behavior is documented in hierarchical order (social > solitary object > solitary locomotor play) at each two-minute scan. Especially solitary play bouts are often a combination of locomotor and object play, however. Using a small data set on all-occurrence play behavior collected from March 2014 to August 2014 during full-day focal follows of immature individuals ranging from 0.3 to 8.6 years of age at both study sites, we find that a mean of 11.3± SD 6% of all solitary play bouts is a combination of locomotor and object play (Suaq: 11.4± 6.1% [N=102 full-day focal follows on 4 individuals]; Tuanan: 10.9±SD 6.2% [N=145 full-day focal follows on 11 individuals]).


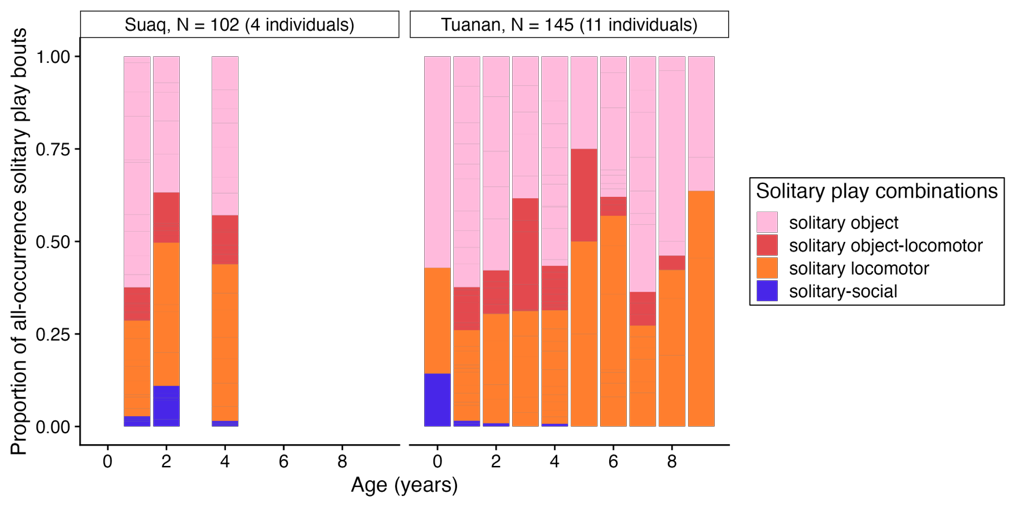


**Figure S1 Proportion of solitary play combinations** by age (years) and study site in immature Bornean (*Pongo pygmaeus wurmbii*, Tuanan, Central Kalimantan, Indonesia, March - August 2014) and Sumatran orangutans (*P. abelii*, Suaq Balimbing, South Aceh, Indonesia, March - August 2014). Figure illustrates all-occurrence, cross-sectional data on solitary play bouts collected during a relatively short period compared to the large data set used for the analyses reported in the main text.

*Distribution of individual focal observations for ontogenetic trajectories*

*
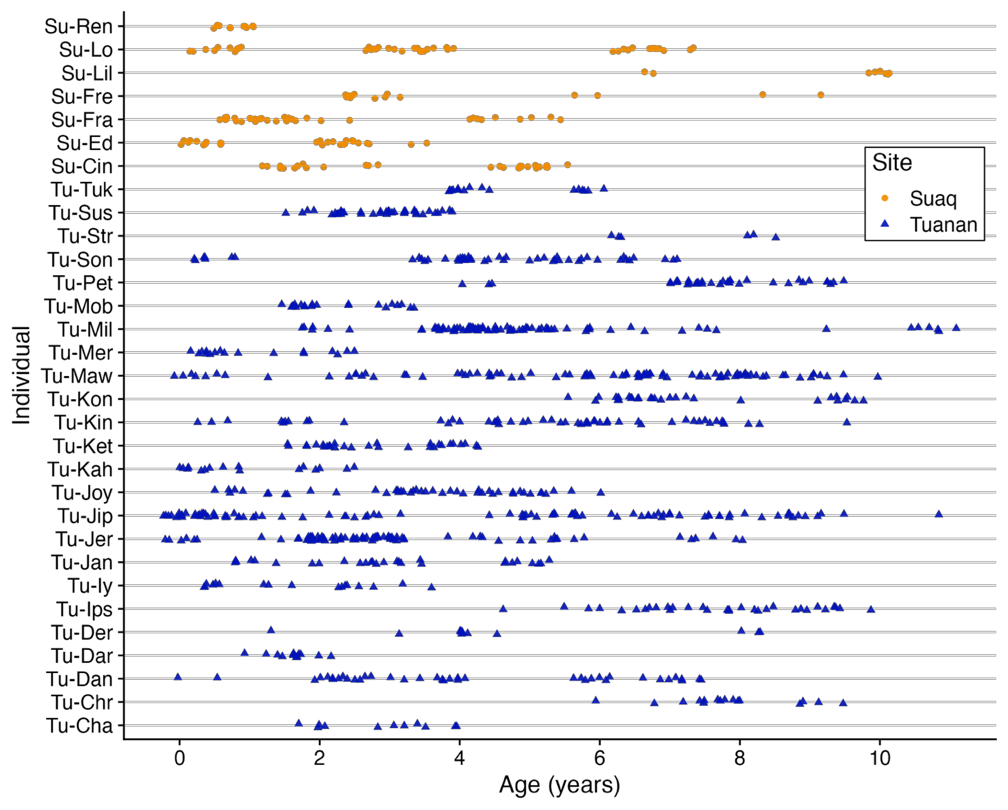
*

**Figure S2 Overview of focal observations by individual** (y-axis), age (years) and study site (Tuanan: *Pongo pygmaeus wurmbii*, Central Kalimantan, Indonesia, 2003 - 2018; Suaq: *P. abelii*, Suaq Balimbing, South Aceh, Indonesia, 2007 - 2018). Each data point illustrates one full-day focal follow for a specific age-individual combination of the data set used for the analyses on ontogenetic play trajectories (N = 1037). A jitter function has been applied to make overlapping data points visible (i.e., at a specific age and during a specific follow period).

# Results

**Table S1 Solitary play frequency:** Effects of individual, social and ecological variables on the daily 2-min play scan count per full-day focal follow by dependent and semi-dependent immature individuals, as obtained from negative binomial GLMMs (N=1037 full-day focal follow days [Tuanan: N=873 (*Pongo pygmaeus wurmbii*, Central Kalimantan, Indonesia, 2003 - 2018); Suaq: N=164 (*P. abelii*, Suaq Balimbing, South Aceh, Indonesia, 2007 - 2018)]). Likelihood ratio test model comparisons to the corresponding null model is indicated below the response variables. Significant fixed effects (P < 0.05) are indicated in bold

| **Variables** | **Estimate ± SE** | **Exp. Coef.** | **95% CI** | **z** | **P** |
| --- | --- | --- | --- | --- | --- |
| Intercept | -2.841 ± 0.309 | 0.06 | [-3.447, -2.235] | - | - |
| ID (N= 31) / Followperiod (N=343) | *random intercept* | |  |  |  |
| Observer (N=49) | *random intercept* | |  |  |  |
| Total 2-min activity scans | *offset (log-link)* | |  |  |  |
| Site (Suaq vs. Tuanan) | 0.014 ± 0.311 | 1.01 | [-0.595, 0.623] | - | - |
| z Age | -0.875 ± 0.268 | 0.42 | [-1.400, -0.350] | - | - |
| z Age^2^ | -0.606 ± 0.301 | 0.55 | [-1.197, -0.016] | - | - |
| **Site : zAge** | -2.070 ± 0.314 | 0.13 | [-2.685, -1.454] | -6.592 | **<0.001** |
| **Site : z Age^2^** | -1.517 ± 0.347 | 0.22 | [-2.198, -0.837] | -4.370 | **<0.001** |
| Sex (female vs. male) | -0.178 ± 0.195 | 0.84 | [-0.561, 0.204] | -0.914 | 0.361 |
| **z FAI** | 0.092 ± 0.046 | 1.10 | [0.001, 0.183] | 1.973 | 0.048 |
| Social play (0/1) | 0.055 ± 0.044 | 1.06 | [-0.030, 0.141] | 1.266 | 0.205 |
| *χ^2^*_5,13_ = 327.64, P<0.0001, ΔAIC = 311.64 | | | | | |

##
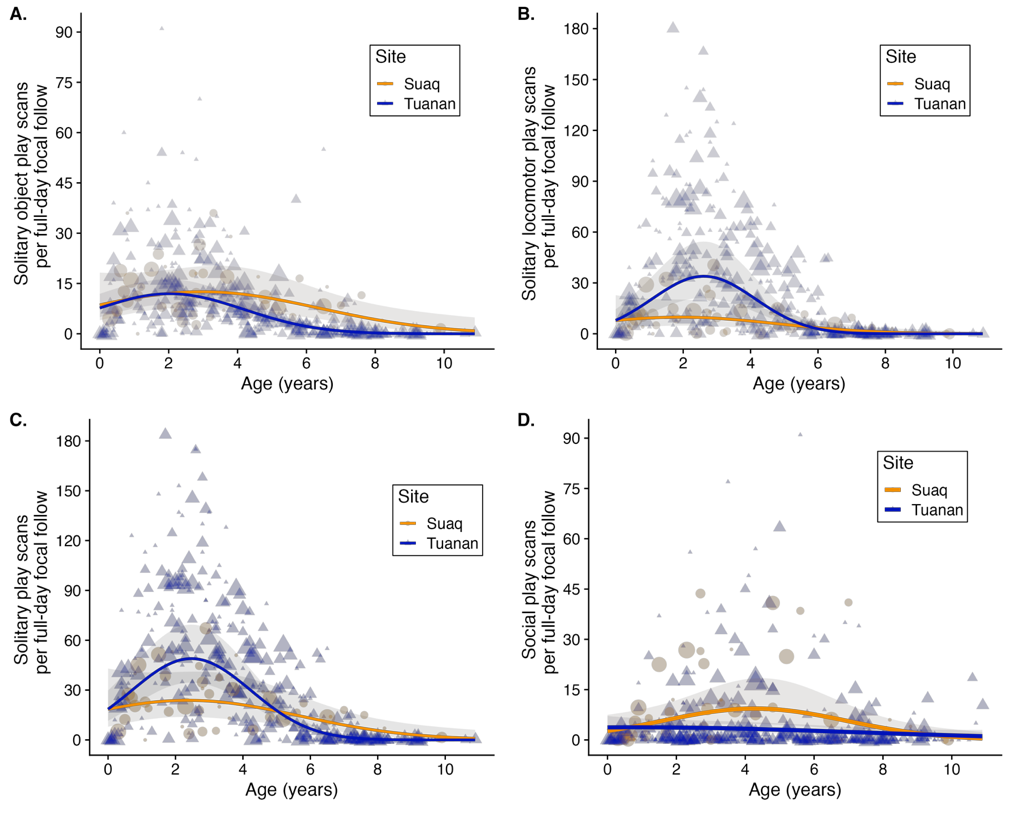


**Figure S3 Play trajectories**: Number of daily 2-min scan samples spent in play behavior by age (years) (x-axis) and study site (Suaq: Sumatran orangutans (*P. abelii*)*,* Suaq Balimbing, South Aceh, Indonesia, 2007-2018; Tuanan: Bornean orangutans (*Pongo pygmaeus wurmbii*)*,* Tuanan, Central Kalimantan, Indonesia, 2003-2018); Data points illustrate mean A. solitary object, B. solitary locomotor, C. all solitary, and D. social play scans per full-day focal follow by individual (N = 31) and follow period (N = 349), whereby data point size is relative to the number of full-day focal follows. The solid lines indicate the model predictions based on the corresponding full models per study site (Table 2b-d) and the shaded area illustrate the 95% confidence intervals. *Note*: Difference in y-axis between figure A and D vs. B and C.

*Frequency of different play types and local fruit availability*


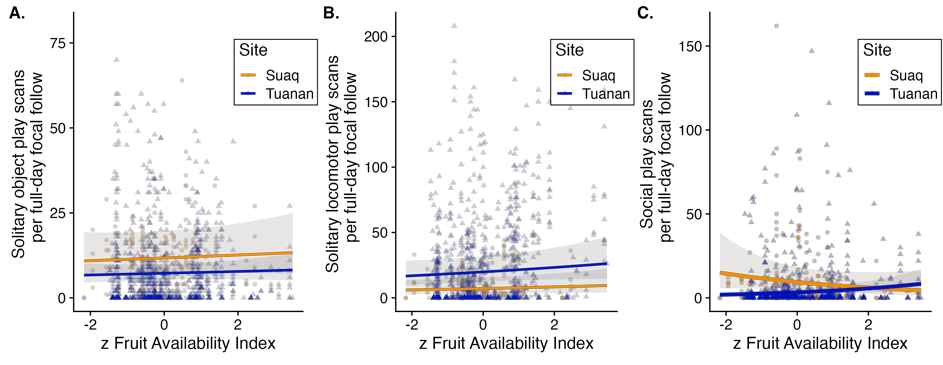


**Figure S4 Play frequency and local fruit availability**: Solitary object (A.), solitary locomotor (B.), and social (C.) play frequency (2-min bouts per full-day focal follow) by z Fruit Availability Index (x-axis) and study site (Suaq: Sumatran orangutans (P. abelii), Suaq Balimbing, South Aceh, Indonesia, 2007-2018; Tuanan: Bornean orangutans (Pongo pygmaeus wurmbii), Tuanan, Central Kalimantan, Indonesia, 2003-2018). Dots illustrate raw data, lines and ribbons show model predictions, and upper and lower confidence intervals as obtained from the models reported in the main manuscript (Table 2). Note y-axes are on different scales for each panel.

*Play opportunities - association frequency*


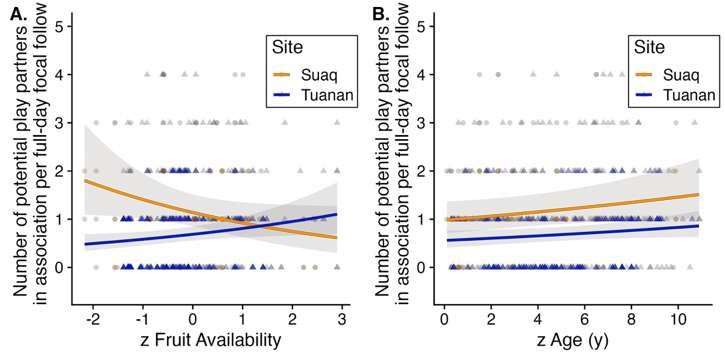


**Figure S5 Number of potential play partners in association** per full-day focal follow by local Fruit Availability (A.) and age (B.) of the focal individual and by study site (Suaq: Sumatran orangutans (P. abelii), Suaq Balimbing, South Aceh, Indonesia, 2007-2018; Tuanan: Bornean orangutans (Pongo pygmaeus wurmbii), Tuanan, Central Kalimantan, Indonesia, 2003-2018). Data points indicate raw data and lines and shaded area the model predictions and confidence intervals. Note: One data point from Suaq is not shown in the figure with 9 play partners in association on one full-day focal follow.

**Table S2 Number of different association partners per day:** Effects of individual, social and ecologic variables on the number of (a) unweaned immatures, (b) weaned immatures, and (c) unflanged males that immatures had in association during a full-day focal follow (N = 731 days) from two study populations (Suaq: Sumatran orangutans (P. abelii), Suaq Balimbing, South Aceh, Indonesia, 2007-2018; Tuanan: Bornean orangutans (Pongo pygmaeus wurmbii), Tuanan, Central Kalimantan, Indonesia, 2003-2018), as obtained by Poisson GLMMs. Significant fixed effects (P < 0.05) are indicated in bold

| **Reponse** | **Variables** | **Estimate ± SE** | **Exp. Coef.** | **95% CI** | **z** | **P** |
| --- | --- | --- | --- | --- | --- | --- |
| **a) Number of dependent immatures in association** | Intercept | -1.216 ± 0.312 | 0.30 | [-1.827, -0.605] |  |  |
|  | ID (N = 29) / FP (N = 261) | *random intercept* |  |  |  |  |
|  | Site (Suaq vs. Tuanan) | 0.303 ± 0.325 | 1.35 | [-0.333, 0.939] | - | - |
|  | z Fruit Availability Index | -0.418 ± 0.207 | 0.66 | [-0.825, -0.012] | - | - |
|  | **Site : z FAI** | 0.545 ± 0.228 | 1.72 | **[0.098, 0.991]** | **2.392** | **0.017** |
|  | **z Age** | 0.615 ± 0.137 | 1.85 | **[0.346, 0.884]** | **4.486** | **< 0.001** |
|  | Sex (female vs. male) | -0.253 ± 0.252 | 0.78 | [-0.747, 0.242] | -1.001 | 0.317 |
|  | Matriline (large vs. small) | -0.521 ± 0.339 | 0.59 | [-1.185, 0.144] | -1.536 | 0.125 |
|  | χ^2^ _3,9_ = 29.86, P < 0.001, ∆AIC = 17.86, R^2^_c_ = 0.22 | | | | | |
| **b) Number of weaned immatures in association** | (Intercept) | -1.563 ± 0.479 | 0.21 | [-2.501, -0.624] | - | - |
|  | ID (N = 29) / FP (N = 261) | *random intercept* |  |  |  |  |
|  | Site (Suaq vs. Tuanan) | -0.948 ± 0.487 | 0.39 | [-1.903, 0.007] | -1.946 | 0.052 |
|  | **z Age** | -0.980 ± 0.202 | 0.38 | **[-1.376, -0.585]** | **-4.856** | **< 0.001** |
|  | Sex (female vs. male) | -0.292 ± 0.445 | 0.75 | [-1.164, 0.579] | -0.658 | 0.511 |
|  | z Fruit Availability Index | 0.070 ± 0.119 | 1.07 | [-0.163, 0.304] | 0.591 | 0.555 |
|  | **Matriline (large vs. small)** | 1.095 ± 0.539 | 2.99 | **[0.039, 2.152]** | **2.032** | **0.042** |
|  | χ^2^ _3,8_ = 31.35, P < 0.001, ∆AIC = 21.35, R^2^_c_ = 0.33 | | | | | |
| **c) Number of unflanged males in association** | Intercept | -0.971 ± 0.295 | 0.38 | [-1.550, -0.393] | - | - |
|  | ID (N = 29) / FP (N = 261) | *random intercept* |  |  |  |  |
|  | **Site (Suaq vs. Tuanan)** | -1.146 ± 0.311 | 0.32 | **[-1.757, -0.536]** | **-3.681** | **< 0.001** |
|  | **z Age** | 0.351 ± 0.160 | 1.42 | **[0.038, 0.665]** | **2.200** | **0.028** |
|  | Sex (female vs. male) | 0.206 ± 0.265 | 1.23 | [-0.312, 0.725] | 0.780 | 0.435 |
|  | z Fruit Availability Index | -0.016 ± 0.110 | 0.98 | [-0.230, 0.199] | -0.144 | 0.886 |
|  | Matriline (large vs. small) | 0.275 ± 0.332 | 1.32 | [-0.376, 0.927] | 0.829 | 0.407 |
|  | χ^2^ _3,8_ = 12.74, P = 0.03, ∆AIC= 2.74, R^2^_c_ =0.24 | | | | | |


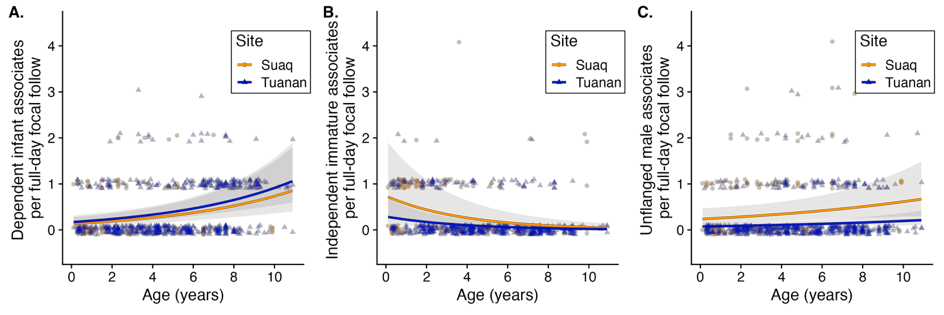


**Figure S6 Number of potential play partners in association** per full-day focal follow by partner age class A. dependent immatures (unweaned infants), B. semi-dependent and independent immatures and C. unflanged males by focal age (x-axis) and by study site (Suaq: Sumatran orangutans (P. abelii), Suaq Balimbing, South Aceh, Indonesia, 2007-2018; Tuanan: Bornean orangutans (Pongo pygmaeus wurmbii), Tuanan, Central Kalimantan, Indonesia, 2003-2018). Data points indicate raw data, i.e., individual full-day focal follow days. Solid lines and shaded areas show model predictions (Table S2), and upper and lower confidence intervals. Note: A vertical jitter function was applied to data points to make overlaying data points more visible.

## *Motivation to play - social play probability and frequency during dyadic association*


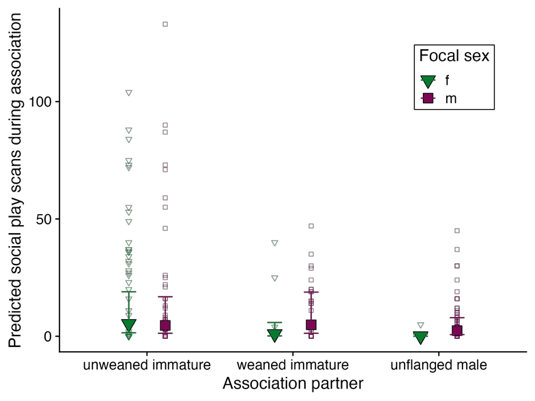


**Figure S7 Social play scans during dyadic associations with different partners** (x-axis) **and by focal sex**. The unfilled data points illustrate the raw data based on daily dyadic association units on full-day focal follows (N = 636); the filled larger points indicate model predictions and the error bars the 95% confidence intervals (as obtained from the model in Table 5c). Note: 30 2-min scans are equivalent to 1 h of focal observation.


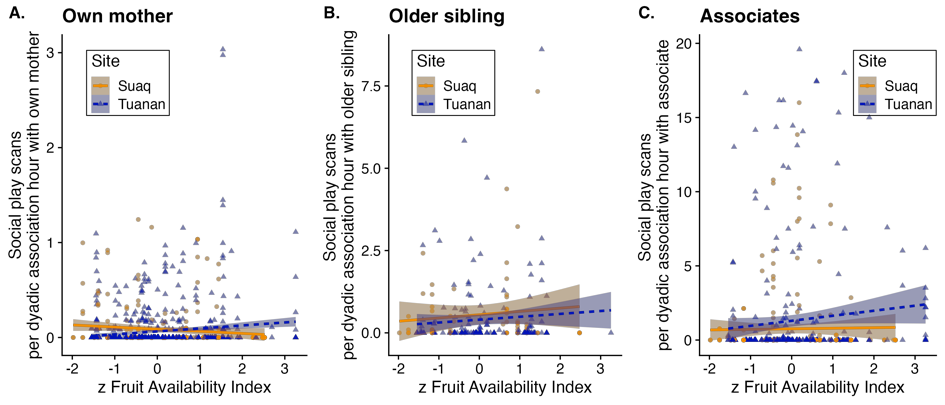


**Figure S8 Social play scans per dyadic association hour during full-day focal follows by z Fruit Availability Index** (x-axis) and study site (Suaq: Sumatran orangutans (P. abelii), Suaq Balimbing, South Aceh, Indonesia, 2007-2018; Tuanan: Bornean orangutans (Pongo pygmaeus wurmbii), Tuanan, Central Kalimantan, Indonesia, 2003-2018). A. Social play with the own mother; B. Social play with the older sibling; C. Social play with associates. Note: Lines illustrate correlation between zFAI and social play scans, not model predictions. Note: 30 2-min scans are equivalent to 1 h of focal observation.


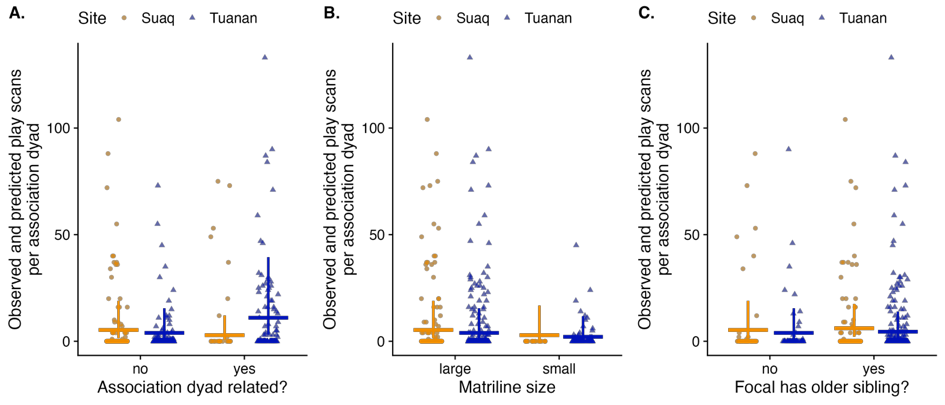


**Figure S9 Number of social play scans per association dyad** (own mother and siblings excluded) and day by maternal relatedness of the dyad (A.), matriline size of the focal (B.), and if the focal has an older sibling (C.) by study site (Suaq: Sumatran orangutans (P. abelii), Suaq Balimbing, South Aceh, Indonesia, 2007-2018; Tuanan: Bornean orangutans (Pongo pygmaeus wurmbii), Tuanan, Central Kalimantan, Indonesia, 2003-2018). The data points indicate raw data based on daily dyadic associations per focal individual. The horizontal lines illustrate model predictions and the vertical lines upper and lower confidence intervals of model predictions. Note: 30 2-min scans are equivalent to 1 h of focal observation.
